# Supplementary material for: Predatory protists reduce bacteria wilt disease incidence in tomato plants
Source: Nat Commun. 2024 Jan 27;15:829. doi: 10.1038/s41467-024-45150-0 (PMC10821857; doi:10.1038/s41467-024-45150-0)
Supplement: Supplementary file 1 — Supplementary Information [file 41467_2024_45150_MOESM1_ESM.pdf]

Supplementary figures

Supplementary Fig. 1. Rhizosphere bacterial (A), fungal (B) and protistan (C) diversities and bacterial (D), fungal (E) and protistan (F) community compositions (based on the Bray–Curtis distance) of different fertilization treatments in the field experiment.

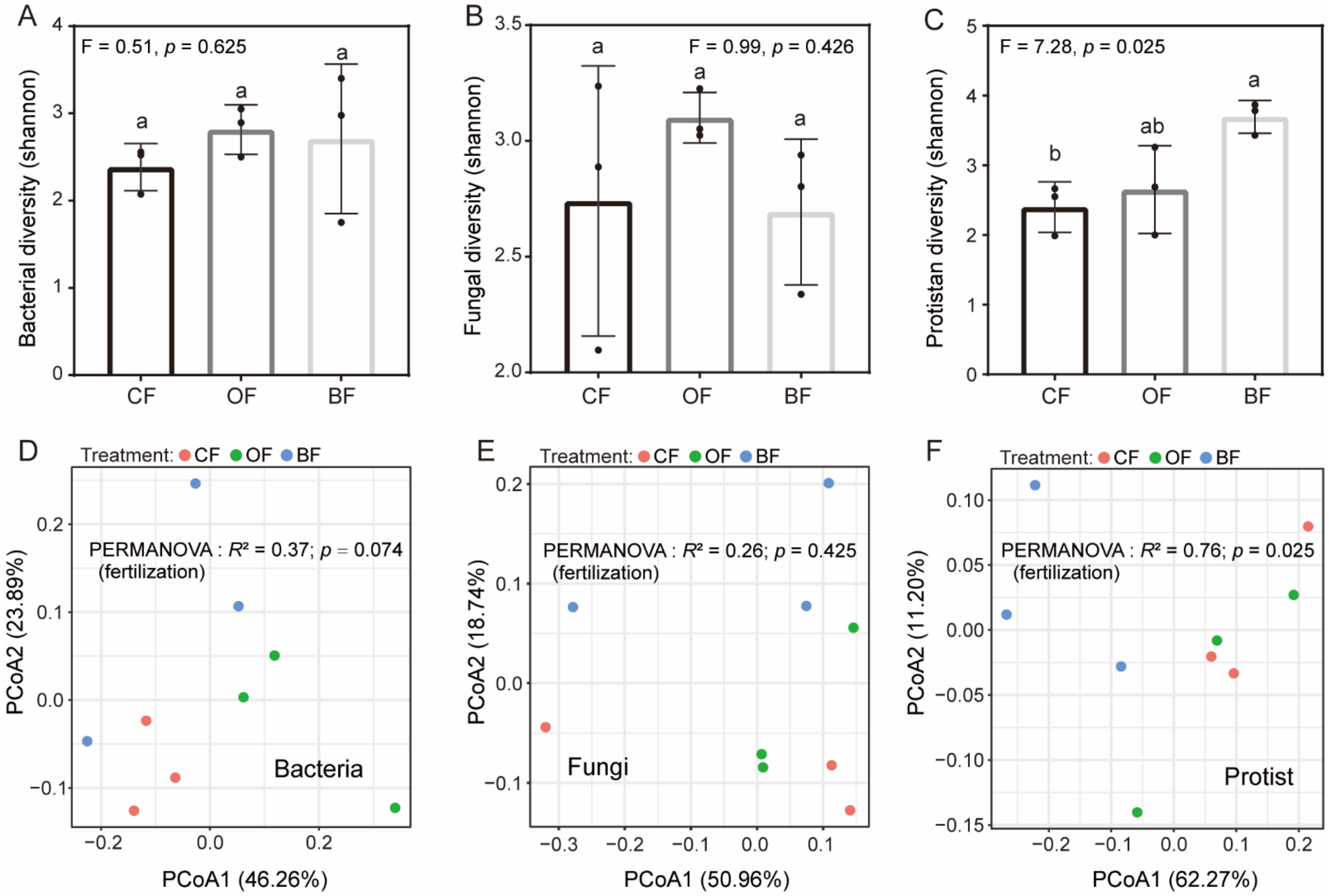

CF: conventional fertilization, OF: organic fertilization, BF: bioorganic fertilization. ANOVA with two-sided Tukey's multiple comparison was used for the statistical analysis. Letters: significant differences between treatments ($p < 0.05$). Results are means ± standard deviation (n = 3 biologically independent samples). Source data are provided as a Source Data file.

Supplementary Fig. 2. Effects of different fertilization regimes on the relative abundances of rhizosphere predatory protists in the field experiment.

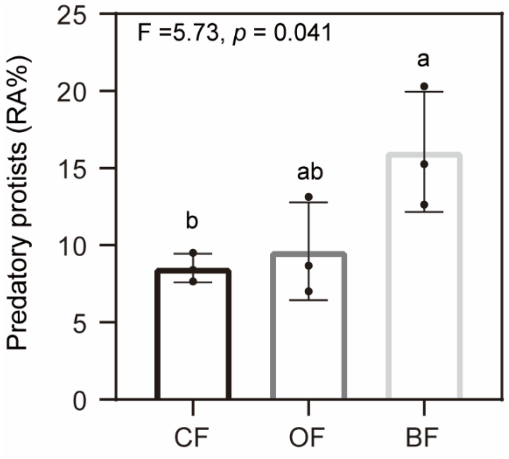

CF: conventional fertilization, OF: organic fertilization, BF: bioorganic fertilization. ANOVA with two-sided Tukey's multiple comparison was used for the statistical analysis. Letters: significant differences between treatments ($p < 0.05$). RA = relative abundance. Results are means ± standard deviation (n = 3 biologically independent samples). Source data are provided as a Source Data file.

Supplementary Fig. 3. Spearman's rank correlation between tomato bacterial wilt disease incidence and the density of rhizosphere *R. solanacearum* in the greenhouse experiment.

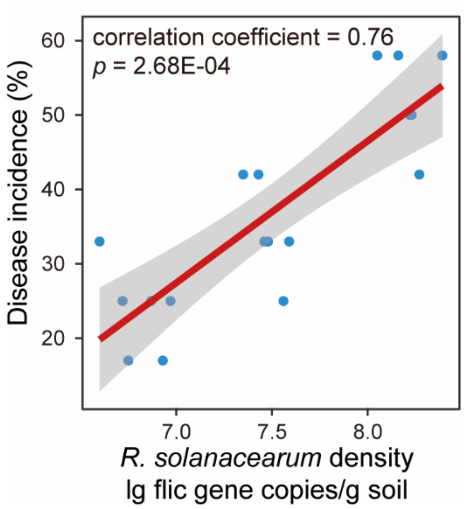

R. solanacearum = *Ralstonia solanacearum.* Two-sided Spearman's correlation was performed to explore the relationship between the density of *R. solanacearum* and disease incidence. Solid lines denote statistically significant ($p < 0.05$). Source data are provided as a Source Data file.

Supplementary Fig. 4. Effects of different concentrations of *Colpoda* on rhizosphere bacterial and fungal diversities (A) and community compositions (B). The rhizosphere bacterial diversities of treatments with different concentrations of *Colpoda* (C).

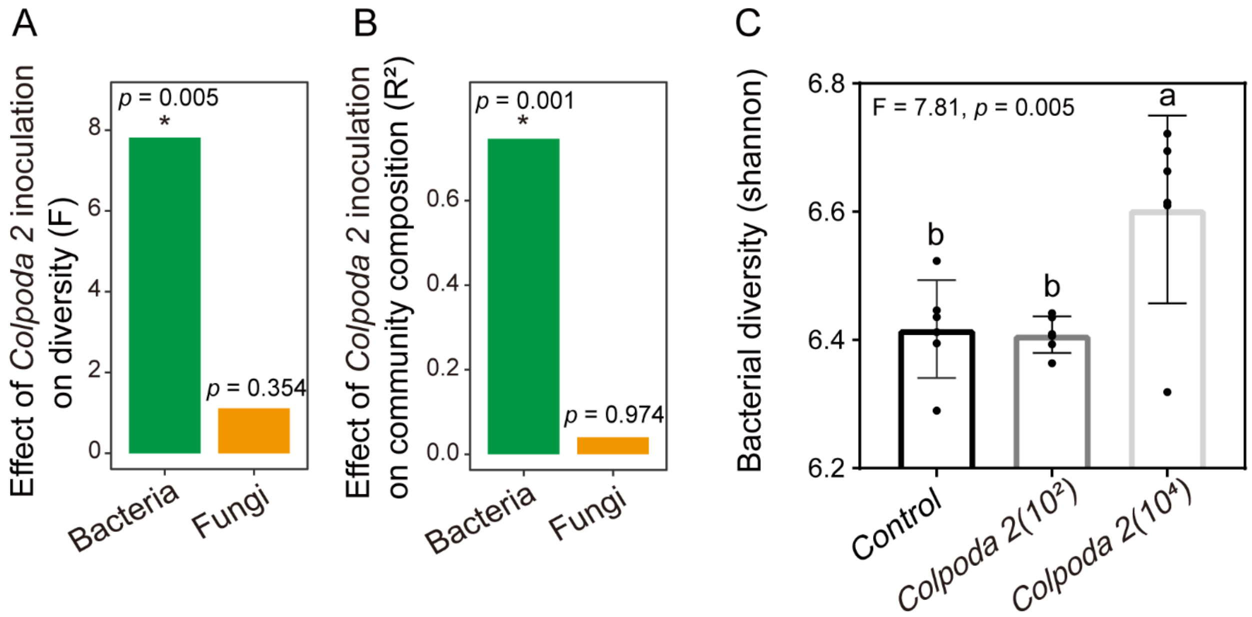

In panel A, ANOVA with two-sided Tukey's multiple comparisons was used for the statistical analysis. Asterisk: significant differences between treatments ($p < 0.05$). In panel B, Permutational multivariate analysis of variance (PERMANOVA) was used for the statistical analysis. Asterisk: significant differences between treatments ($p < 0.05$). In panel C, ANOVA with two-sided Tukey's multiple comparison was used for the statistical analysis. Letters: significant differences between treatments ($p < 0.05$). Results are means ± standard deviation (n = 6 biologically independent samples). Source data are provided as a Source Data file.

Supplementary tables

Supplementary Table 1. Spearman's rank correlation between the relative abundances of protistan functional groups and tomato bacterial wilt disease incidence.

	disease incidence
predators	***p* = 0.007**
parasites	*P* = 0.798
saprotrophs	*P* = 0.058
plant pathogens	*P* = 0.077
phototrophs	*P* = 0.088

Two-sided Spearman's correlation was performed to explore the relationship between the relative abundances of protistan functional groups and tomato bacterial wilt disease incidence.

Supplementary Table 2. Spearman's rank correlation coefficient between the relative abundance of *Colpoda* (P_OTU67) and the density of *R. solanacearum*.

	The relative abundance of *Colpoda* (P_OTU67)
R. solanacearum density	correlation coefficient = -0.93, *p* = 2.36E-04

Two-sided Spearman's correlation was performed to explore the relationship between the relative abundance of *Colpoda* (P_OTU67) and the density of *R. solanacearum*.

Supplementary Table 3. Detailed information for *Colpoda* strains based on GenBank.

Colpoda ID	Taxonomy annotation (GenBank)	Sequence ID (GenBank)	The percentage of similarity (GenBank)
Colpoda 1	*Colpoda inflata strain*	KJ607918.1	99.41%
Colpoda 2	*Colpoda inflata strain*	KJ607918.1	99.35%

Supplementary Table 4. The sequence similarity of *Colpoda* strains and P_OTU 67.

Colpoda ID	The sequence similarity with P_OTU 67
Colpoda 1	98.43%
Colpoda 2	99.71%

Supplementary Table 5. Spearman's rank correlation coefficient between the relative changes of bacteria density in different bacteria + *Colpoda* treatments and the relative changes of *R. solanacearum* density in different bacteria + *R. solanacearum* treatments.

	The relative changes of bacteria density in different bacteria + *Colpoda* treatments
The relative changes of *R. solanacearum* density in different bacteria + *R. solanacearum* treatments	correlation coefficient = -0.73, *p* =1.99E-15

Two-sided Spearman's correlation was performed to explore the relationship between the relative changes of bacteria density in different bacteria + *Colpoda* treatments and the relative changes of *R. solanacearum* density in different bacteria + *R. solanacearum* treatments.

Supplementary Table 6. Detailed fertilization scheme of the field experiment.

Treatment	Fertilization scheme
CF (conventional fertilization)	120 kg ha^{-1} nitrogen (N), 180 kg ha^{-1} phosphorus (P) and 120 kg ha^{-1} potassium (K) mineral fertilizers were applied to the soil in each season
OF (organic fertilization)	7500 kg ha^{-1} organic fertilizer (1.75% nitrogen (N), 0.82% phosphorus (P) and 1.42% potassium (K) in 2018) was applied
BF (bioorganic fertilization)	7500 kg ha^{-1} bio-organic fertilizer (1.85% nitrogen (N), 0.80% phosphorus (P) and 1.46% potassium (K) in 2018) was applied

Supplementary Table 7. Detailed information for the quantitative PCR.

Target group	Forward primer sequence (5´-3´)	Reverse primer sequence (5´-3´)	Reference or source
R. solanacearum	GAACGCCAACGGTGCGAACT	GGCGGCCTTCAGGGAGGTC	1
Pseudomonas	GAGTTTGATCCTGGCTCAG	GAGTTTGATCCTGGCTCAG	2
Lysobacter	GAGCCGACGTCGGATTAGCTGTT	AAGGAGGTGWTCCARCC	3
Streptomyces	GAACTGAGACCGGCTTTTTGA	GGTGGCGAAGGCGGA	4
Arthrobacter	GCTGGTTTGAGAGGACGACCAGC	AGCCCATGACGTTTCTTTCCT GCCA	5
Chitinophaga	TTRAAGATGGSYGTGCRYC	CGCTACATGACATATTCCGCT	6

Supplementary References

1. Schönfeld, J., Heuer, H., van Elsas, J. D. & Smalla, K. Specific and sensitive detection of Ralstonia solanacearum in soil on the basis of PCR amplification of fliC fragments. *Appl. Environ. Microbiol.* **69**, 7248–7256 (2003).
2. Tao, C. *et al.* Bio-organic fertilizers stimulate indigenous soil Pseudomonas populations to enhance plant disease suppression. *Microbiome* **8**, 137 (2020).
3. Hu, Y. Detection methods for the genus Lysobacter and the species Lysobacter enzymogenes. (University of Nebraska - Lincoln, 2010).
4. Li, C., Tian, Q., Rahman, M. K. u. & Wu, F. Effect of anti-fungal compound phytosphingosine in wheat root exudates on the rhizosphere soil microbial community of watermelon. *Plant Soil* **456**, 223–240 (2020).
5. Ruiz, O. N. *et al.* Metagenomic characterization reveals complex association of soil hydrocarbon-degrading bacteria. *Int. Biodeterior. Biodegrad.* **157**, 105161 (2021).
6. Hayes, A. C., Liss, S. N. & Allen, D. G. Growth kinetics of Hyphomicrobium and Thiobacillus spp. in mixed cultures degrading dimethyl sulfide and methanol. *Appl. Environ. Microbiol.* **76**, 5423–5431 (2010).
